# Determination of Hexapeptide ALA-ASP-LEU-LYS-PRO-THR by MALDI MS

**DOI:** 10.1007/s10989-012-9334-8

**Published:** 2012-11-25

**Authors:** Anna Olejnik, Izabela Nowak, Krystian Eitner, Grzegorz Schroeder

**Affiliations:** Faculty of Chemistry, Adam Mickiewicz University in Poznań, Umultowska 89b, 61-614 Poznań, Poland

**Keywords:** Hexapeptide, ALA-ASP-LEU-LYS-PRO-THR, Mass spectrometry, Matrix MALDI MS

## Abstract

The hexapeptide ALA-ASP-LEU-LYS-PRO-THR is currently widely used as an active ingredient in commercially available creams. Therefore, the proper analytical procedure to detect this compound is a very important issue. This paper presents the determination of the hexapeptide in solution and in cosmetic formulations by matrix assisted laser desorption ionisation mass spectrometry. We developed a procedure for the selective binding of the low molecular weight peptide by using a two-component matrix which enabled us to characterize the hexapeptide directly without any initial processing. Furthermore, the extensive computer simulations were carried out to assist in this analysis of the MS spectra.

## Introduction

The tremendous progress in cosmetic science has created a great variety of the new skincare products with innovative and highly sophisticated active ingredients. Bioactive peptides are a group of these ingredients that have been widely used in the skincare and pharmaceutical industry. Creams including peptides are the new cosmeceuticals with the potential to improve the appearance of aging skin and are known as anti-age or anti-wrinkle cosmetics. There are number of reasons for the recent sudden growth in interest in bioactive peptides. The most significant is their activity. Peptides are involved in many natural processes with relevance to skincare such as the cell migration, modulation of cell proliferation, angiogenesis, melanogenesis and protein synthesis and regulation (Fields et al. 2009; Zhang and Falla 2009). Peptides used in the cosmetic industry can be divided into three main categories depending on their functions—signal peptides, neurotransmitter-affecting peptides and carrier peptides (Lupo 2005; Lupo and Cole 2007). Signal peptides have the ability to increase fibroblast production of collagen or decrease collagenase breakdown of existing collagen therefore improve the appearance of the fine and coarse wrinkles (Lupo and Cole 2007). Neurotransmitter were developed to mimic the botulinum neurotoxins. Their effect is connected with their ability to block the entrance of calcium ions, and thus muscular contraction is attenuated. The neuropeptides transfer the signal to skin cells to inhibit muscle movement. They can potentially decrease facial muscle contraction, reducing lines and wrinkles (Blanes-Mira et al. 2002). Carrier peptides stabilize and deliver important trace elements such as copper necessary for wound healing and enzymatic processes (Lupo and Cole 2007).

Most of peptides used in cosmetic products are specially cleaved fragments of larger precursor molecules such as proteins. This means that the creation of bioactive peptides is based on the amino acid sequences of their natural equivalents. These types of peptides have a sequence of between 2 and 20 amino acids and are called biomimetic because they mimic the structure of certain regions of a protein in the extracellular matrix of the dermis, such as α1-pro-collagen, α2-pro-collagen 1, elastin, tropoelastin, fibronectin, or lamin-5 (Denommee, US Pat. No. 2007/0237735). The biomimetic peptides are used in cosmetic formulations in a concentration ranging between about 0.5 and 10 % by weight.

The hexapeptide with the sequence ALA-ASP-LEU-LYS-PRO-THR represents a group of biomimetic fibronectin peptides, and it is created in order to mimic fibronectin, a protein present in the extracellular matrix. This protein, which is synthesized by fibroblasts and keratinocytes, is involved in many biological processes, including tissue repair and cell migration such as in embryonic migratory pathways and in the provisional matrix of healing wounds and cell adhesion (Yamada 2000). The good adhesion is linked with an appropriate three-dimensional organization of cutaneous tissue that is connected with healthy and young skin. It is thought that increasing fibronectin levels help to improve the appearance of aging skin. Fibronectin is crucial for the maintenance of skin integrity and important for skin repair, its level is increased during wound healing process. However, it was found that UV radiation increases its degradation in skin. Furthermore, fibronectin was found at decreased levels in the papillary dermis of aging skin (Ptchelintser, Avon Products Inc., US Pat. No. 2009/0082252; Ray et al. 2006; Labat-Robert et al. 2000). Due to the size of fibronectin, its effectiveness at the cutaneous level is limited. Therefore the hexapeptide (ALA-ASP-LEU-LYS-PRO-THR) is created to play the role of a homolog of a 6-amino acid sequence present in the type III unit of the fibronectin molecule and thus can mimic the activities of above-mentioned protein. This peptide stimulates an extracellular matrix protein synthesis in vivo, promotes the adhesion between skin cells in order to provide curative or preventive treatment of ageing skin symptoms and thus improves skin appearance (Dal Farra, Domloge, US Pat. No. 2004/0141939). The application of this hexapeptide on cultured keratinocytes reinforces cell adhesion. Moreover, the hexapeptide has the ability to increase integrin expression and provide a signal for skin repair. The preferred concentration range of the peptide with the sequence ALA-ASP-LEU-LYS-PRO-THR in a cosmetic composition is between 0.5 and 3.0 % by weight (Denommee, US Pat. No. 2007/0237735). The potential anti-age effects of a synthetic fibronectin-like peptide was investigated in two double blind studies. A detailed questionnaire given to volunteers and clinical studies of treated areas enabled to assess the effects of active ingredient. In the first study twelve volunteers applied the cream containing the fibronectin-like peptide twice a day on the back of one hand and the placebo on the other hand. A lightening effect and increased smoothness of skin was observed after 1 h, 3 h and after 7 days. In the second study one group of volunteers applied cream containing the active ingredient on the lips, and the other group applied placebo. A significant improvement of skin hydration (155.56 % at 3 h) and smoothness (425.01 % at 1 h and 533.33 % at 3 h) was observed. Therefore the researchers suggest that the fibronectin-like peptide can be of great use in anti-aging skin care products (Farra et al. 2007). The synthetic peptide was developed by Vincience and is commercially known as Vinci 02. The company carried out and extensive research on the effects of this compound and they concluded that Peptide Vinci 02 was highly stable and had a high fibronectin-like effect on cell adhesion, cell differentiation (Cosmetics and Toiletries 2006).

The low molecular peptides are currently widely used as an active ingredient in commercially available creams. Thus the proper qualitative analysis of these compounds is a very important issue. The hexapeptide ALA-ASP-LEU-LYS-PRO-THR was chosen as an exemplary peptide widely used in cosmetic industry. This article presents the determination of hexapeptide both in commercial available solution and in cosmetic formulation by matrix assisted laser desorption ionisation (MALDI) mass spectrometry. We developed a procedure for the selective binding of the low molecular weight peptide by using a two-component matrix which enabled us to determine the hexapeptide directly without any initial processing. The appropriate methodology and the data interpretation will be presented in details. In order to assist the MS analyses the extensive computer simulations were carried out.

## Experimental

### Materials

The peptide ALA-ASP-LEU-LYS-PRO-THR solution (Peptide Vinci 02) was obtained from IPS Vincence. The solution consisted of water, butylene glycol (30–60 %) and the hexapeptide (0.5–3 % by weight). In order to prepare cream the following materials were used as received: Creagel^®^ EZ 7, Polyacrylamide, Hydrogenated Polydecene, Laureth-7 (Creations Couleurs, France), Alphaflow^®^ 20, Hydrogenated Polydecene (Creations Couleurs, France).

### Method for Cream Preparation

1.5 g of Creagel^®^ EZ (instant emulsifiers and emulsion stabilizers) was mixed with 2.5 g of Alphaflow, then water was added gradually while mixing until an emulsion was obtained. The preparation was carried out at room temperature. Vinci solution was added to the prepared emulsion under continuous mechanical stirring.

### MALDI Mass Spectrometry

The mass spectrometric experiments were performed using a Waters/Micromass (Manchester, UK) Q-TOF Premier mass spectrometer (software MassLynx V4.1, Manchester, UK) fitted with a 200 Hz repetition rate Nd/YAG laser (*λ* = 355 nm, power density 10^7^ W/cm^2^). Argon was used as a collision gas at flow rates of 0.5 ml/min in the collision. The samples were prepared by mixing the sample in methanol with a matrix. After a few minutes at room temperature, the spot was dry and mass spectra could be recorded.

### Determination of the Relative Stabilities of Complexes

In order to determine the relative stabilities of complexes we measured the MS spectra as a function of collision energy. The relative intensity of the signals was determined from the ratio I/Imax. The relative stability of the peptide complex with the ions was determined from the value of d(I/Imax)/d collision energy.

### Theoretical Calculations

Peptide structure was generated using the molconvert program (Marvin 5.3.6, 2010 from ChemAxon http://www.chemaxon.com) from fasta string “ADLKPT”. Structure was used to descriptors calculation with Padel Descriptors Software based on CDK (chemistry development kit) utility. A MOPAC2009 (Stewart 2008) was used for calculations, and a new RM1 (Rocha et al. 2006) Hamiltonian was applied to optimize geometries without any constraints. The criteria for terminating all optimizations, electronic and geometric, were increased by a “precise” keyword.

## Results and Discussion

**Fig. 1:**
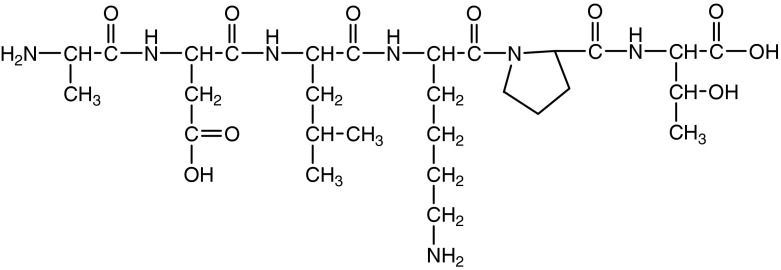
The chemical structure of the hexapeptide

The hexapeptide ALA-ASP-LEU-LYS-PRO-THR is now widely used as an active ingredient in commercial creams. The amino acid sequences ALA-ASP-LEU and PRO-THR are particularly effective in physiological processes (Siemion et al. 1990). The chemical structure of the hexapeptide with the sequence ALA-ASP-LEU-LYS-PRO-THR is shown in the Fig. 1.

### The Theoretical Study

**Fig. 2:**
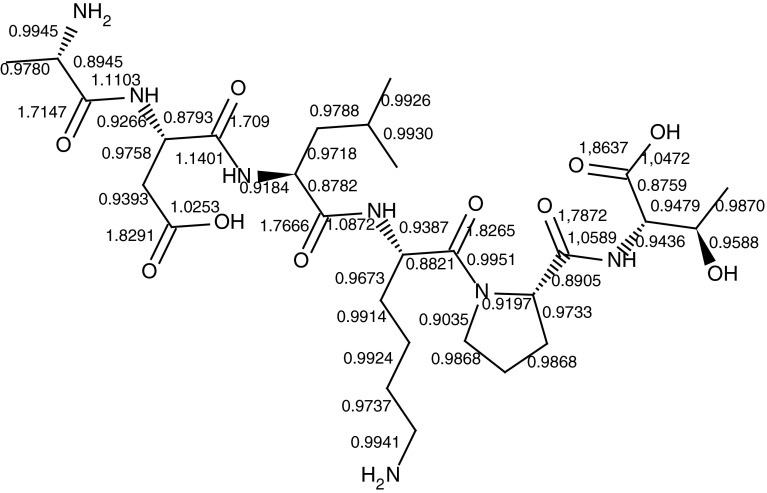
Bond orders for the ALA-ASP-LEU-LYS-PRO-THR peptide structure (aliphatic hydrogen atoms were neglected)

The theoretical calculations were used to determine bond orders were used to determine bond orders. Figure 2 and Table 1 present bond orders for the ALA-ASP-LEU-LYS-PRO-THR peptide structure (aliphatic hydrogen atoms were neglected).

**Table 1:** The values of bond order for ALA-ASP-LEU-LYS-PRO-THR peptide bonds

	Number of bonds	1	2	3	4	5
	1st ALA-ASP	0.8954	1.7147	1.1103	0.8529	0.9266
	2nd ASP-LEU	0.8793	1.7090	1.1401	0.8367	0.9184
	3rd LEU-LYS	0.8782	1.7666	1.0872	0.8382	0.9387
	4th LYS-PRO	0.8821	1.8265	0.9951	None	0.9197
	5th PRO-THR	0.8905	1.7872	1.0589	0.8616	0.9436

The theoretical calculations enabled us to determine the bond order and thus it helps to indicate the stability of the bonds. These data were helpful in interpretation of fragments in MS/MS spectrum (Fig. 4).

### MS Study

**Fig. 3:**
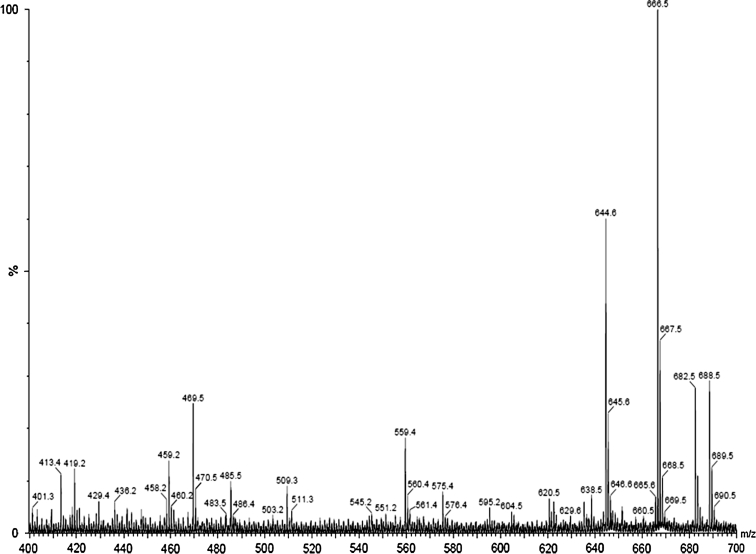
MALDI mass spectrum of hexapeptide in commercially available solution (Vinci 02); *m*/*z* 644.6 (M+H)^+^, *m*/*z* 666.5 (M+Na)^+^, *m*/*z* 682.5 (M+K)^+^, *m*/*z* 688.5 (M+2Na)^+^

In this paper we describe our strategy to identify peptides in the commercially available solution and in cosmetic formulation using MALDI mass spectroscopy. In the MALDI-TOF spectrum, the signals were registered as a function of intensity. Initially we focused our attention on analyzing peptide in the solution. In the Fig. 3 the spectrum of the hexapeptide (MW = 643.7) obtained using 2,5-dihydroxybenzoic acid (DHB) as a matrix is presented. The positive ion mode of the hexapeptide (TOF MS LD+) shows a protonated molecular ion [M+H]^+^ at *m*/*z* 644.6 and a molecular ion with an associated sodium [M+Na]^+^ at *m*/*z* 666.5 with the relative intensity ratio 1:2. Two additional ions of hexapeptide were observed at *m*/*z* 682.5 (M+K)^+^, *m*/*z* 688.5 (M+2Na)^+^.

**Fig. 4:**
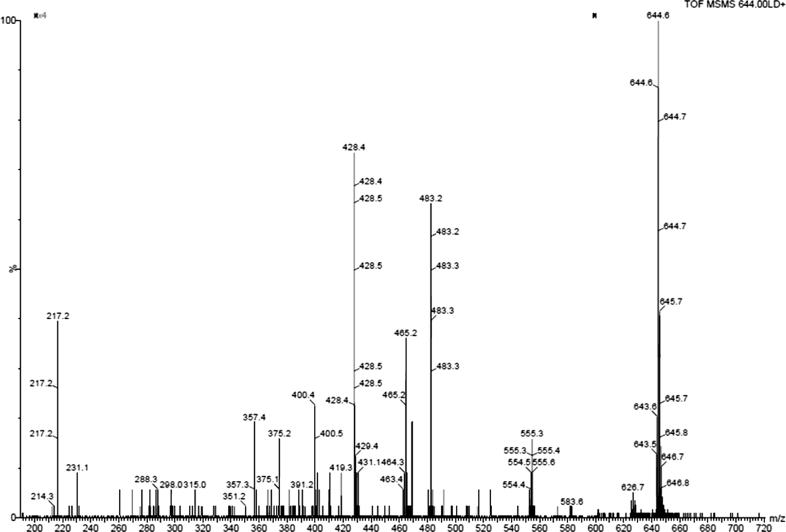
MS/MS spectrum of the hexapeptide in commercially available solution (Vinci 02)

**Fig. 5:**
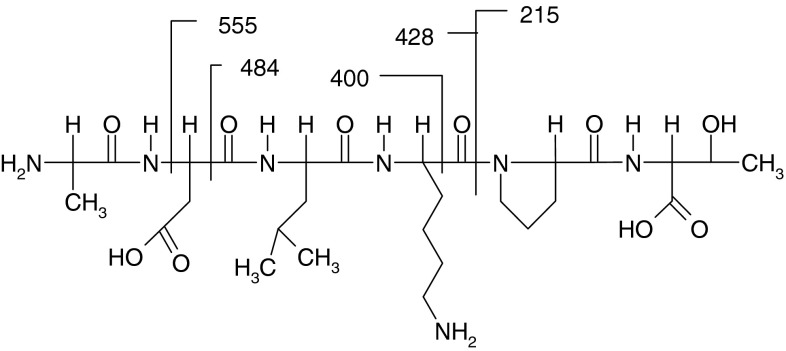
MS/MS fragmentation pattern of the hexapeptide

Peptide structural data and its aminoacid sequence can be obtained by collisional activation of selected singly or multiply charged precursor ions. Tandem mass spectrometry (MS/MS) enabled us to determine the main peptide ions in the fragmentation process. The proper interpretation of peptide fragmentation is a key element in the analytical process. The product ions that are observed in spectrum (Fig. 4) can be found at *m*/*z* 555.4, 483.2, 465.2 (483.2-H_2_O), 428.4 and 217.2. The interpretation of these fragments is presented in the Fig. 5.

Fragmentation of peptides occurs at the peptide bonds (N–C and C–CO) that have the lowest bond orders in these molecules. The correlation between bond orders and the location of fragmentations is a helpful tool in the interpretation of MALDI MS spectra. The bond order value characterizes the bond energy therefore the theoretical calculations were useful to determine the fragmentation pattern.

**Fig. 6:**
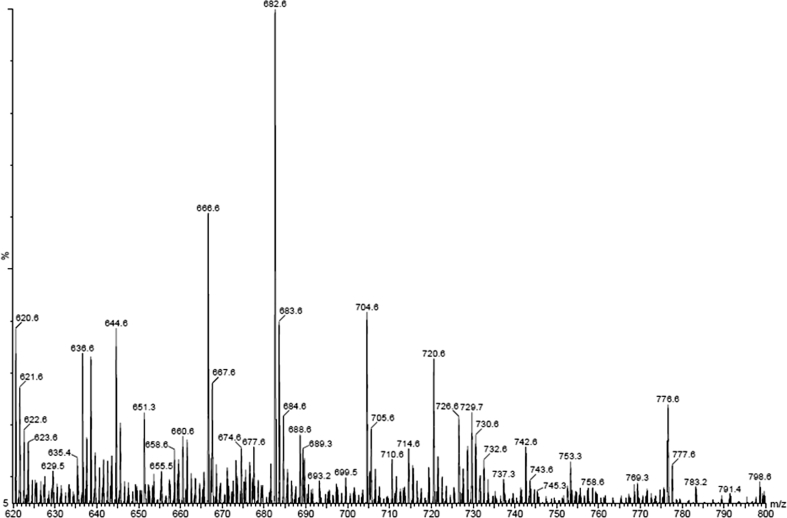
MALDI MS spectrum of equimolar solutions containing lithium, sodium, potassium, rubidium and cesium with the hexapeptide (DHB as the matrix) *m*/*z* 644.6 (M+H)^+^, 651.3 (M+Li)^+^, 658.6 (M+Li)^+^, 666.6 (M+Na)^+^, 682.6 (M+K)^+^, 688.6 (M+2Na)^+^, 720.6 (M+2K)^+^, 729.7 (M+Rb)^+^, 776.6 (M+Cs)^+^

Furthermore, we found that the investigated peptide was able to form complex ions with alkali metals. The MALDI MS spectrum of equimolar solutions containing cations of lithium, sodium, potassium, rubidium and cesium with an excess of hexapeptide is presented in the Fig. 6.

**Fig. 7:**
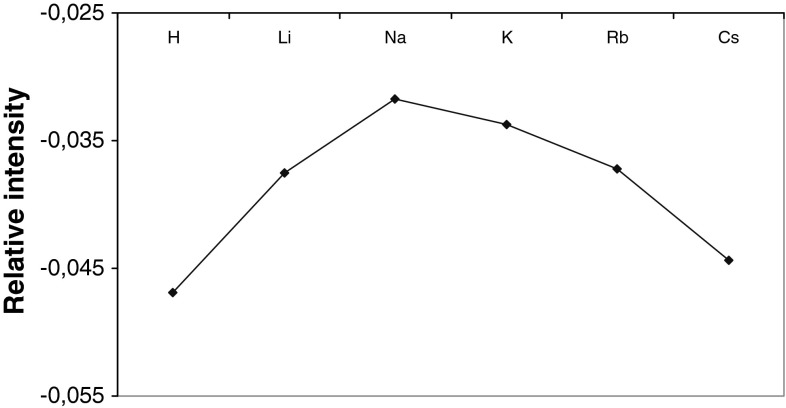
The hexapeptide preferences to form complex ions calculated from MALDI MS measurements

**Fig. 8:**
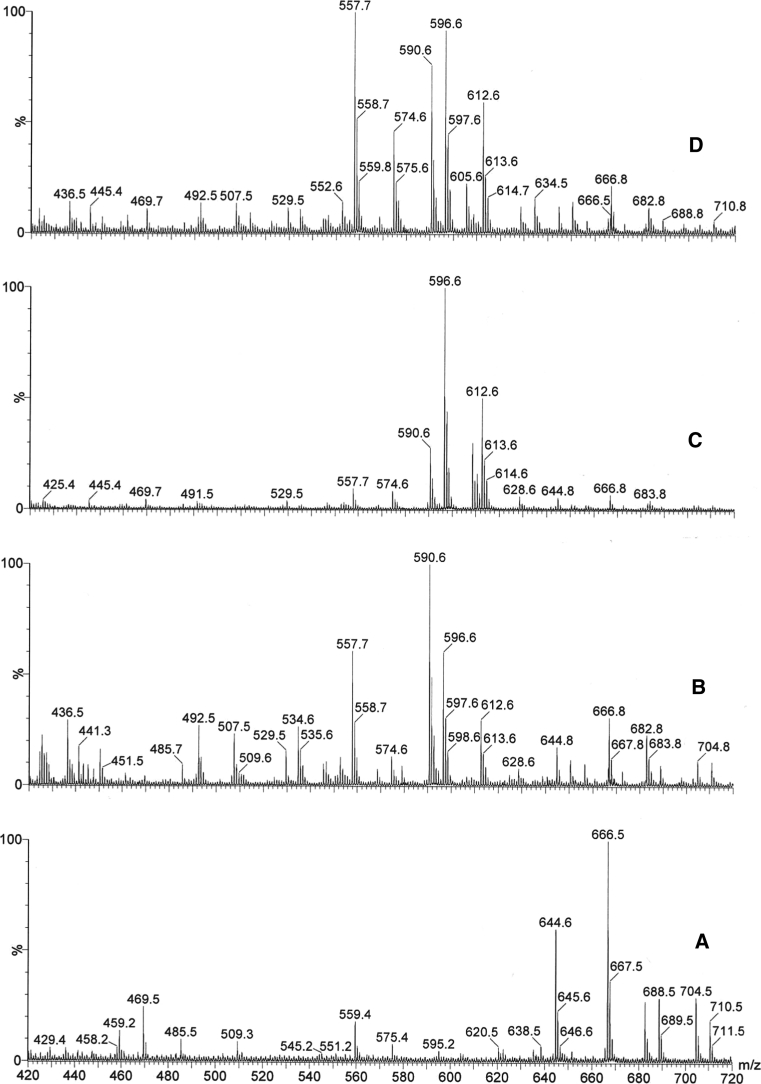
MALDI MS spectra: **a** hexapeptide without cream (matrix–DHB), **b** cream with hexapeptide (two-component matrix–mesoporous material SBA-15 L64 material and DHB, in ratio 1:1), **c** cream with hexapeptide (two-component matrix: carbon nanotubes and DHB, in ratio 1:1), **d** cream with hexapeptide (two-component matrix: OH-functionalized multi-walled carbon nanotubes and DHB, in ratio 1:1)

The hexapeptide preferences to form complex ions with alkali metals were determined by measurements of the signal intensity and ionization energy (ce) of the investigated complexes. The results obtained are presented in the Fig. 7. The affinity of the peptide for cations decreased as follow Na^+^ > K^+^ > Li^+^ > Rb^+^ > Cs^+^ > H^+^. These results give us information about the peptide preferences to form complex ions. This can be useful to understand better the reason why there are mostly ion associated with sodium in the spectrum of peptide in cosmetic formulation (that will be presented in Fig. 8).

In the second part of our work, we focused our attention on identifying the hexapeptide in cosmetic formulations. The qualitative analysis of peptides in cosmetics formulations and body fluids seems to be really challenging, especially if the assay is made without any initial processing such as separation or extraction of the determined component. However, the usage of MALDI mass spectroscopy with assisted matrices enabled us to characterized the hexapeptide directly without any initial processing.

The concept of using the additional compound to immobilize classic MALDI matrix materials for the analysis of small molecules such as dopamine (153.08 Da) and serotonin (176.09 Da). was demonstrated by Mullens et al. (2011). The new matrix materials were based on silica gel and mesoporous silica, SBA-15. It was hypothesized that due to the larger surface area and spot uniformity the modified mesoporous silica facilitated the ionization process. The SBA-15 functionalized with quinoline moiety was also successfully applied as a matrix in the MALDI-TOF–MS analysis of small molecules such as saccharides, amino acids—l-arginine, metabolites, and natural honey. Compared with DHB and SBA-15, the modified SBA-15 exhibited several advantages in the analysis of small molecules (as with MALDI-TOF–MS, such as less background interference ions, high homogeneity, and better reproducibility (Li et al. 2009).

In order to accomplish our studies, we have developed a procedure for the selective binding of low molecular weight peptides by using a two-component matix consisting of the classical matrix-DHB and a supplementary material such as:

1. mesoporous material SBA-15 L64-mesopores diameter 4.3 nm, 23 % micropores, surface area 800 m^2^/g.
2. carbon nanotubes-diameter 10–20 nm, length 10–30 μm, SSA 200–350 m^2^/g.
3. OH-functionalized multi-walled carbon nanotubes-diameter 10–20 nm, length 10–30 μm, SSA 200 m^2^/g, mole fraction of surface carbon atoms functionalized with –OH: 21–25 mol%.

The spectra of the hexapeptide obtained using different two-component matrices are presented in the Fig. 8. In all cases we could identify the hexapeptide as an ion associated with sodium at *m*/*z* 666.8. Depending on the associated matrix, the intensity of this signal was different.

## Conclusion

The determination of the hexapeptide by MALDI mass spectrometry was carried out successfully. The theoretical calculations enabled us to determine bond orders that were helpful to understand the fragmentation process in mass spectroscopy. The hexapeptide was easily identified in commercial available solution, however in cosmetic formulation the use of the additional matrix was required. The application of the two-component matrixes enabled us to identify the hexapeptide in cosmetic formulation without any initial processing. It was demonstrated that the hexapeptide’s affinity to form complexes with alkali metals is the highest for sodium.
